# Identification of *BRAF* V600E Mutation in Cerebrospinal Fluid Aids in Diagnosing Leptomeningeal Carcinomatosis Arising From Pleomorphic Xanthoastrocytoma: A Case Report

**DOI:** 10.1002/cnr2.70487

**Published:** 2026-03-18

**Authors:** Randy S. D'Amico, Patricia Avancena, Alon Kashanian, Rosivel Galvez, Deborah Gruber, Sami Saba, Avraham Zlochower, Manju Harshan, Morana Vojnic

**Affiliations:** ^1^ Department of Neurosurgery Lenox Hill Hospital, Northwell Health New York New York USA; ^2^ Division of Hematology/Oncology, Lenox Hill Hospital Northwell Health New York New York USA; ^3^ Department of Neurosurgery Northwell Health New Hyde Park New York USA; ^4^ Department of Neurology Lenox Hill Hospital, Northwell Health New York New York USA; ^5^ Department of Radiology Lenox Hill Hospital, Northwell Health New York New York USA; ^6^ Department of Pathology Lenox Hill Hospital, Northwell Health New York New York USA; ^7^ Rutgers Cancer Institute of New Jersey New Brunswick New Jersey USA

## Abstract

**Background:**

Pleomorphic xanthoastrocytomas (PXAs) are rare primary central nervous system (CNS) tumors that appear heterogeneous on imaging and histology and typically cause headaches or seizures on initial presentation. Alongside high rates of favorable prognosis after surgical excision exist similarly high rates of recurrence. Leptomeningeal spread on recurrence is even rarer and more challenging to diagnose.

**Case:**

We describe a case of a 40‐year‐old man with a history of surgically resected PXA presenting 12 years later with persistent headaches and lower back pain. Imaging studies revealed arachnoiditis, and a subsequent brain biopsy was nondiagnostic. Serial CSF studies only revealed the presence of atypical cells too few to further characterize via standard histology studies, with rare small lymphocytes and monocytoid cells. Submitting these cells for next‐generation sequencing ultimately revealed a BRAF V600E mutation typically found in PXAs, thereby confirming the diagnosis of leptomeningeal recurrence and revealing a therapeutic target.

**Conclusion:**

This case highlights the utility of next‐generation sequencing as a means of non‐invasively diagnosing leptomeningeal disease in recurrent PXA and potentially in other cancer types as well.

## 1 Introduction

Pleomorphic xanthoastrocytomas (PXAs) are rare CNS neoplasms, primarily affecting younger individuals. Initially described in 1973 and recognized as WHO grade II tumors, PXAs generally show a favorable prognosis with a 75% five‐year survival rate [1]. However, they carry a significant risk of recurrence (78%) [2] and may progress to aggressive WHO grade III anaplastic forms (A‐PXA). Notably, leptomeningeal dissemination (LMD) is a rare but serious complication, typically emerging in advanced stages.

PXAs exhibit variable imaging features and notable histological pleomorphism. Genetic mutations, particularly BRAF V600E [3, 4, 5], are frequently observed and can inform diagnosis. Surgical resection remains the primary treatment, with limited benefit from chemotherapy and radiotherapy. This paper presents a novel case of PXA LMD diagnosed 12 years post‐surgical intervention through next‐generation sequencing of cerebrospinal fluid (CSF) tumor cells, underscoring the utility of molecular testing in diagnosing LMD. This approach represents a significant advancement in the non‐invasive diagnostic capabilities for complex cases.

## 2 Informed Consent

This case report was reviewed by the Northwell Health Institutional Review Board and determined to be exempt from full review as a single‐patient case report. Consent for publication was obtained from the patient's next of kin.

## 3 Methods

CSF was collected via lumbar puncture and ventricular drain and processed within 2 h. Samples were spun down to isolate cells and cell‐free supernatant. Tumor DNA was extracted and analyzed using next‐generation sequencing via a CLIA‐certified laboratory platform. Variant annotation and filtering were performed using institutional pipelines. BRAF p.V600E c.1799T>A was detected at 17% variant allele frequency. Histologic slides were processed with standard H&E and immunohistochemistry protocols. MRI scans were performed on a 3 T Siemens system with T1, T2‐FLAIR, and post‐contrast T1‐weighted sequences.

## 4 Case Description

**FIGURE 1:**
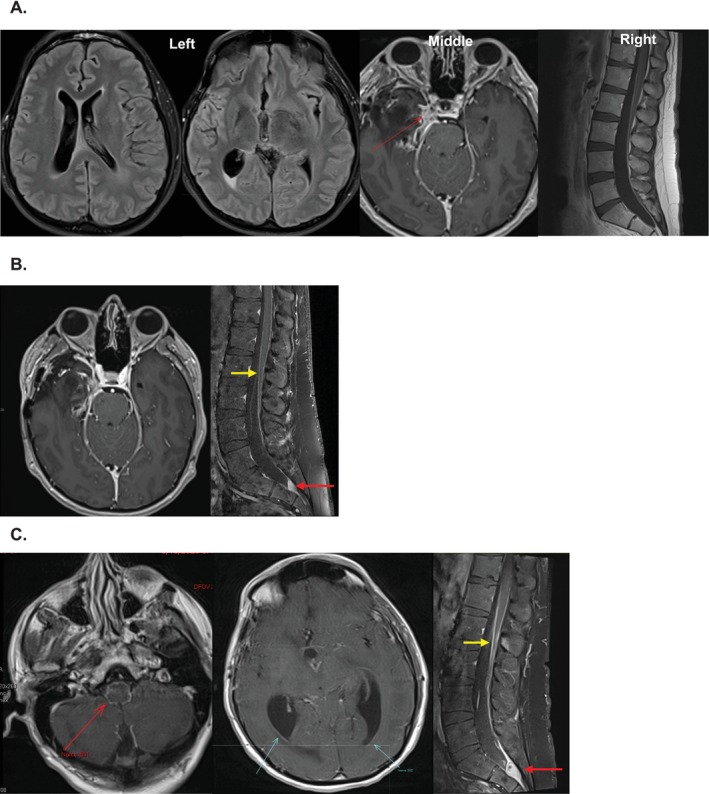
(A) Pre‐treatment MRI: Left: axial T2‐FLAIR images demonstrating enlarged lateral and third ventricles consistent with hydrocephalus. Periventricular edema notable surrounding the right occipital horn. Middle: axial post contrast image with nodular leptomeningeal enhancement in the right parasellar region (arrow). Right: Sagittal post contrast T1‐weighted images of lumbar spine with peripheral location of nerve roots and mild nerve root enhancement of the cauda equina nerve roots consistent with arachnoiditis. (B) Post‐treatment MRI: Left—at 4 months treatment: Follow‐up axial post contrast demonstrating stable nodular enhancement in the right parasellar region. Right—axial post‐contrast T1‐weighted image of the lumbar spine demonstrating linear leptomeningeal enhancement (yellow arrow) and a thecal sac metastasis (red arrow). (C) MRI showing progression at 7 months of treatment: Left and middle—Axial post contrast images demonstrating leptomeningeal enhancement surrounding the brainstem and ependymal enhancement of the bilateral occipital horns. Right—Sagittal post contrast images of the lumbar spine demonstrating progressive leptomeningeal enhancement of the lower thoracic spinal cord (yellow arrow), the cauda equina, and nodular intradural disease at the sacrum (red arrow).

**FIGURE 2:**
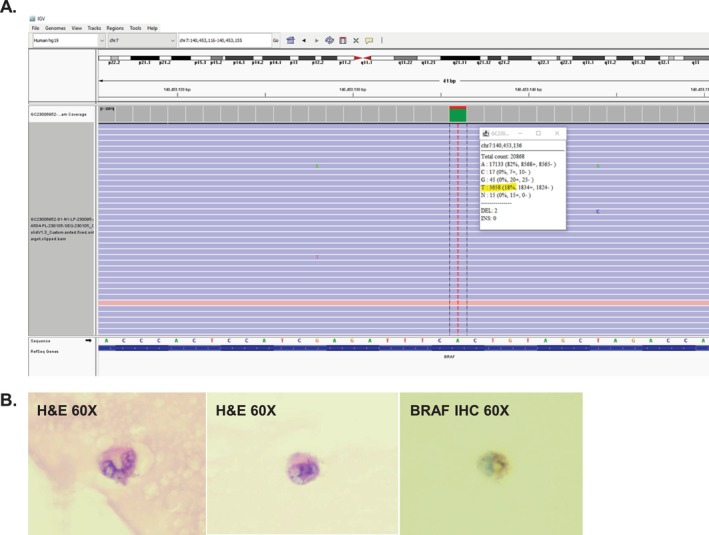
(A) Next generation sequencing of *BRAF* in CSF tumor cell revealed *BRAF* p.Val600Glu c.1799T>A, Exon 15, detected at 17% allele frequency (VAF). (B) Representative microscopy images 60× magnification for visualized tumor cells with H&E and IHC for BRAF.

A 40‐year‐old male with a history of supratentorial pleomorphic xanthoastrocytoma (PXA) underwent gross total resection of the tumor 12 years prior. Following surgery, he remained asymptomatic and was successfully tapered off antiepileptic medications. Interval follow‐up appointments document normal neurologic exams and the absence of concerning symptoms for the subsequent 12 years. In 2022, he presented to the emergency department at Lenox Hill Hospital with persistent headaches and severe low back pain that had developed over the preceding 4 months. The patient underwent MRI of the craniospinal axis which suggested arachnoiditis‐induced hydrocephalus (Figure 1A). A lumbar puncture revealed elevated opening pressure (60 mmHg H20; reference range 4.4–14.7 mmHg), atypical cells, low glucose (29 mg/mL; reference range 45–80 mg/mL), and high protein (69 mg/dL; reference range 15–45 mg/dL). The patient underwent placement of an external ventricular drain, which provided symptom relief, and simultaneously underwent right frontal convexity brain biopsy which was non‐diagnostic. Serial CSF analysis revealed persistent atypical cells with no definitive malignant features. However, due to relative acellularity, standard immunohistochemistry on the atypical cells was not feasible. There were scattered lymphocytes, but no eosinophils or plasma cells, suggesting a non‐inflammatory process. Interestingly, single gene testing of the isolated atypical cells for BRAF identified a V600E mutation, confirming leptomeningeal dissemination (LMD) of the patient's previous PXA (Figure 2A), subsequently also confirmed by IHC staining for BRAF (Figure 2B).

Before discharge, a ventriculoperitoneal shunt was placed. Treatment commenced with temozolomide (TMZ) and BRAF‐MEK inhibitors dabrafenib/trametinib, but the latter was discontinued after one dose due to pyrexia and hypotension. Following five cycles of TMZ, MRIs indicated stable disease (Figure 1B). However, the patient was readmitted 2 months later with worsening neck and back pain and evidence of recurrent communicating hydrocephalus. Shunt evaluation revealed elevated protein in the CSF and a shunt malfunction, leading to ventriculostomy replacement. Dabrafenib/trametinib was reattempted but discontinued after 8 days due to severe pyrexia amid a concurrent COVID infection. Dabrafenib/trametinib was chosen based on institutional availability and precedent in PXA literature. Given the patient's intolerance, dabrafenib monotherapy or alternative agents such as tovorafenib were considered but not pursued due to rapid clinical deterioration. The patient's condition deteriorated despite ventricular drainage, and subsequent MRI revealed significant leptomeningeal disease progression (Figure 1C). Craniospinal radiation was deferred due to the diffuse disease burden and the patient's poor functional status at the time of progression. Confronted with these complications and treatment intolerance, the patient transitioned to hospice care and expired soon thereafter, approximately 12 years after initial diagnosis.

## 5 Discussion

The majority of PXAs (85%–94%) harbor a homozygous or hemizygous deletion of *CDKN2A/B* and 60%–78% show an activating *BRAF* V600E mutation, which is associated with better outcomes and is less prevalent in more aggressive A‐PXAs [4, 5, 6]. In addition, TERT promoter mutations in recurrent or anaplastic PXAs indicate that these alterations are later events linked to high recurrence rates and short recurrence‐free survival [5].

First identified in 2002 by Davies and colleagues [7], BRAF V600E is most commonly present in hairy cell leukemia (nearly 100%), papillary thyroid cancer (70%), melanomas (42%), and Langerhans cell histiocytosis (39%) [8]. It acts by activating the downstream MEK/ERK pathways and leads to cell division and proliferation. The development of targeted therapies like BRAF inhibitors vemurafenib, dabrafenib, and encorafenib, used in combination with MEK inhibitors trametinib, binimetinib, and cobimetinib due to inherent bypass mechanisms to single BRAF inhibition, can influence treatment responses and outcomes.

In our case, molecular testing of CSF derived tumor cells was pivotal in diagnosing LMD of the patient's PXA. This approach underscores the diagnostic utility of CSF‐derived tumor cell sequencing, even when traditional immunohistochemical methods are untenable due to acellularity or other limitations. Furthermore, this case lends support to the use of cfDNA to detect actionable mutations in CNS malignancies, particularly in challenging cases like LMD where obtaining viable tumor tissue is difficult.

Despite *BRAF* V600E being a potential therapeutic target, treatment with BRAF‐MEK inhibitors in our patient was complicated by side effects and concurrent conditions. This reflects the broader challenge in treating *BRAF* V600E mutant tumors, where responses to targeted therapy can vary significantly, and resistance development is common [9, 10].

Our study was limited by a lack of information about our patient's initial tumor diagnosis 12 years ago, including unavailability of neuroaxis imaging and irretrievable details regarding histological and molecular markers from that time. Despite this, this study demonstrates the value of molecular analysis in PXA in providing diagnostic and therapeutic insights, particularly in complex cases with limited biopsy options. Whether or not NGS can fully replace immunohistochemistry in differentiating CNS malignancies is still under debate given that much of the WHO criteria relies heavily on histology to distinguish between low‐ and high‐grade lesions. The *BRAF* V600E mutation, for example, is present in a spectrum of CNS tumors ranging from papillary craniopharyngiomas to pleomorphic xanthoastrocytomas to anaplastic astrocytomas [11, 12]. Indeed, in rarer entities such as PXA, we are still far from having distinct diagnostic molecular signatures able to accurately differentiate among the various cancer subtypes. However, in the accumulation of sequencing data from cfDNA obtained in cases such as our patient's with recurrent PXA manifesting as LMD, we stand to gain invaluable insight via minimally invasive diagnostics.

## 6 Conclusions

We describe the first documented case of PXA LMD diagnosed using next generation sequencing. This non‐invasive method of molecular testing identified a *BRAF* V600E mutation, aiding in assessing disease progression and guiding subsequent targeted combination therapy. Future research is essential to validate this diagnostic tool for LMD in PXAs and potentially other cancer types, enhancing our understanding and management of these complex cases.

## Author Contributions

Conceptualization: R.S.D., P.A., D.G., M.H., M.V.; methodology/validation/supervision: R.S.D., M.V.; investigation: R.S.D., P.A., R.G.; visualization: R.S.D., P.A., A.K., R.G., A.Z., M.H., M.V.; writing/review/editing: R.S.D., P.A., A.K., R.G., D.G., S.S., A.Z., M.H., M.V.

## Funding

The authors have nothing to report.

## Disclosure

M.V.: Consulting or advisory role: AstraZeneca, Bristol‐Meyers Squibb, MJH Life Sciences, IDEOLogy Health, I3 Health, Jannsen, Telix Pharmaceuticals. P.A., A.K., R.G., D.G., S.S., A.Z., M.H., R.S.D. have no disclosures.

## Conflicts of Interest

The authors declare no conflicts of interest.

## Data Availability

Data sharing not applicable to this article as no datasets were generated or analyzed during the current study.
